# InsuTAG: A novel physiologically relevant predictor for insulin resistance and metabolic syndrome

**DOI:** 10.1038/s41598-017-15460-z

**Published:** 2017-11-09

**Authors:** Rohith N. Thota, Kylie A. Abbott, Jessica J. A. Ferguson, Martin Veysey, Mark Lucock, Suzanne Niblett, Katrina King, Manohar L. Garg

**Affiliations:** 1 Nutraceuticals Research Program, School of Biomedical Sciences & Pharmacy, University of Newcastle, Newcastle, NSW 2308 Australia; 2 School of Medicine & Public Health, University of Newcastle, Newcastle, NSW 2308 Australia; 3 School of Environmental & Life Sciences, University of Newcastle, Newcastle, NSW 2308 Australia

## Abstract

The aim of this study was to investigate whether a novel physiologically relevant marker, InsuTAG (fasting insulin × fasting triglycerides) can predict insulin resistance (IR) and metabolic syndrome (MetS). Data of 618 participants from the Retirement Health and Lifestyle Study (RHLS) were evaluated for the current study. IR was defined by homeostatic model assessment (HOMA-IR) scores. Pearson correlations were used to examine the associations of InsuTAG with HOMA-IR and other markers. Predictions of IR from InsuTAG were evaluated using multiple regression models. Receiver operating characteristic curves (ROC) were constructed to measure the sensitivity and specificity of InsuTAG values and to determine the optimum cut-off point for prediction of IR. InsuTAG was positively correlated with HOMA-IR (r = 0.86; p < 0.0001). InsuTAG is a strong predictor of IR accounting for 65.0% of the variation in HOMA-IR values after adjusting for potential confounders. Areas under the ROC curve showed that InsuTAG (0.93) has higher value than other known lipid markers for predicting IR, with a sensitivity and specificity of 84.15% and 86.88%. Prevalence of MetS was significantly (p < 0.0001) higher in subjects with InsuTAG values greater than optimal cut-off value of 11.2. Thus, InsuTAG appears to be a potential feasible marker of IR and metabolic syndrome.

## Introduction

The rise in sedentary lifestyle and ready access to calorie-dense processed foods in the modern-day world has created a convenient environment for the development of complex metabolic abnormalities in humans. Insulin, secreted by the pancreas, is one of the key anabolic hormones that tightly regulates glucose and lipid homeostasis^1^. To maintain this homeostasis, insulin supresses hepatic glucose production, enhances glucose uptake in muscle and fat tissues, increases lipogenesis and regulates hepatic transport of very low-density lipoprotein (VLDL) associated triglycerides (TG)^1^. Chronic over-nutrition results in disruption of insulin signalling pathways leading to increased hepatic secretion of VLDL and possible decrease in the clearance of TG rich lipoproteins. As a result, levels of circulating TG are increased^2^, and this is commonly observed in insulin resistance (IR) and metabolic syndrome^3^. Insulin-mediated suppression of hepatic glucose production is still preserved in IR, however, suppression of VLDL-TG secretion is less pronounced^4^, indicating a rise in TG which may represent an early manifestation of IR. Cross-sectional studies^5,6^ and mechanistic studies^7^ have shown a positive association between circulating TG levels and both insulin sensitivity and action. Kriketos *et al*. 2003 has shown that both skeletal muscle TG and circulating TG were inversely associated with whole body insulin sensitivity^7^. Mingrone *et al*. 1997 showed reversibility of IR by lowering plasma TG in obese individuals with diabetes^8^; together these observations suggest that circulating TG may serve as a marker of IR and its associated complications.

Evidence of a relationship between TG and IR has escalated following the identification of “metabolic syndrome” (MetS), a condition representing a cluster of metabolic abnormalities with central obesity and IR postulated as core components^9–11^. A considerable body of evidence suggests that obesity-associated IR is an independent risk factor and a central component in the pathogenesis of type 2 diabetes and cardiovascular disease (CVD), both of which have reached epidemic proportions worldwide^12,13^. Evidence that IR causes type 2 diabetes comes from cross-sectional studies demonstrating the presence of IR in a majority of type 2 diabetes patients, and prospective studies demonstrating the development of IR long before the onset of type 2 diabetes^14,15^. Dyslipidaemia associated with IR increases the risk of developing CVD in type 2 diabetes^16,17^. It is therefore important to quantify IR and identify MetS for primary and secondary prevention of these metabolic diseases.

Recognition of the importance of IR have prompted the derivation of a number of indices and surrogate markers to quantify IR^18,19^. Among these, the hyperinsulinemic euglycaemic clamp (HEC) technique has been described as the gold standard, providing a direct measure of IR^20^. However, cost, expertise, and the requirement for intravenous insulin infusions and frequent blood sampling limits the application of HEC in epidemiological studies and routine clinical investigations. Homeostatic model assessment (HOMA) is a simple minimally invasive model that predicts IR using fasting steady-state glucose and insulin levels and that has been shown to be highly correlated with clamp insulin sensitive index values^21^. However, HOMA does not consider the level of blood lipids, such as TG or HDL-Cholesterol (HDL-C), despite lipid availability in circulation having an important role in IR and its metabolic complications. In the current study we propose and evaluate a novel tool for estimating IR, InsuTAG. This model is based on the product of fasting blood insulin and fasting blood lipid (TG) levels. InsuTAG is unique in its incorporation of a measure of hyperinsulinemia and a measure of circulating TG in the general population, for identification of IR and its metabolic complications.

## Results

After screening the 618 participants recruited for the RHLS study with exclusion criteria, 486 participants were included in the analysis. One hundred and thirteen participants were excluded because of self-reported diabetes. An additional 19 participants were excluded because they had blood glucose levels ≥7 mmol/L (n = 9); self-reported use of oral anti-hyperglycaemic agents (n = 4); had no blood glucose values recorded (n = 3); and/or self-reported taking TG lowering medications (n = 3). Participant characteristics are outlined in Table 1. The study population was predominantly Caucasian (n = 469, 96.50%), included more females (n = 283, 58.23%) than males (n = 203, 41.77%), had a mean ± SD age of 77.78 ± 7.16 years, and a mean ± SD body mass index (BMI) of 28.05 ± 4.64 kg/m^2^. A total of 82 (16.87%) participants were categorised as IR according to HOMA-IR ≥2.5 and 167 (37.03%) were identified as having MetS according to the International Diabetes Federation (IDF) criteria. Fasting glucose, insulin, TG, weight, waist circumference (WC) and BMI were significantly (p < 0.0001) higher in individuals with IR compared with insulin-sensitive individuals (Table 1). InsuTAG scores ranged from 0.55 to 132.07 across the whole participant group with a median (25^th^–75^th^ percentile) InsuTAG score of 7.22 (4.04–11.33)

**Table 1:** Participant characteristics of all participants and for participants stratified into subgroups by insulin resistance status.

	All Participants n = 486		Insulin Sensitive n = 404		Insulin Resistant n = 82		*p*-value
**Gender (n, (%))**							
Male	203	(41.77)	172	(42.57)	31	(37.80)	0.425*
Female	283	(58.23)	232	(57.43)	51	(62.20)	0.425*
Age (years)	77.78	±7.16	78.03	±7.24	76.54	±6.62	0.085
Waist Circumference (cm)	97.03	±12.73	95.21	±12.06	106.34	±12.03	<0.0001
Height (m)	1.63	±0.09	1.63	±0.09	1.63	±0.10	0.837
Weight (kg)	74.37	±14.59	72.36	±12.81	84.28	±14.35	<0.0001
BMI (kg/m^2^)	28.05	±4.64	27.29	±4.36	31.76	±4.18	<0.0001
Metabolic Syndrome^†^ (n, (%))	167	(37.03)	112	(29.55)	55	(76.39)	<0.0001
**Ethnicity (n, (%))**							
Caucasian	469	(96.50)	392	(97.03)	77	(93.90)	0.160^‡^
Aboriginal/Pacific Islander	6	(1.23)	5	(1.24)	1	(1.22)	0.998^‡^
Asian	1	(0.21)	0	(0.00)	1	(1.22)	0.026^‡^
Don’t know/didn’t respond	10	(2.06)	7	(1.73)	3	(3.66)	0.261^‡^
Dietary Energy Intake (Cal/day)	1971	±725	1968	±735	1983	±679	0.865
Protein (g/day)	90.82	±35.20	90.87	±35.63	90.58	±33.25	0.946
Fat (g/day)	68.95	±31.87	69.42	±32.89	66.67	±26.46	0.478
Saturated fat (%Energy/day)	10.69	±3.24	10.73	±3.38	10.50	±2.43	0.562
Fibre (g/day)	31.81	±14.90	31.99	±15.25	30.91	±13.08	0.549
**Physical Activity** ^**§**^ **(n, (%))**							
High	120	(25.16)	108	(27.20)	12	(15.00)	0.020^‡^
Moderate	263	(55.14)	213	(53.65)	50	(62.50)	0.141^‡^
Low	74	(15.51)	62	(15.62)	12	(15.00)	0.888^‡^
Sedentary	20	(4.19)	14	(3.53)	6	(7.50)	0.102^‡^
Systolic Blood Pressure (mmHg)	145.99	±20.90	146.00	±21.68	145.94	±15.99	0.984
Diastolic Blood Pressure (mmHg)	74.64	±9.70	74.30	±9.64	76.58	±9.91	0.090
Fasting Glucose (mmol/L)	5.22	±0.55	5.14	±0.51	5.64	±0.54	<0.0001
Fasting Insulin (μIU/L)	6.0	(4.0–8.6)	5.4	(3.8–7.2)	13.1	(12.0–16.8)	<0.0001^||^
HbA1c (%)	5.72	±0.34	5.70	±0.31	5.85	±0.44	0.0002
HbA1c (mmol/mol)	39	±3.7	39	±3.4	40	±4.8	0.0002
Total Cholesterol (mmol/L)	4.69	±1.02	4.72	±1.01	4.57	±1.08	0.241
Triglycerides (mmol/L)	1.15	(0.86–1.55)	1.10	(0.83–1.45)	1.54	(1.01–2.00)	<0.0001^||^
LDL-C (mmol/L)	2.58	±0.91	2.58	±0.91	2.53	±0.94	0.645
HDL-C (mmol/L)	1.49	(1.23–1.74)	1.53	(1.29–1.77)	1.25	(1.06–1.54)	< 0.0001^||^
Total-C/HDL-C	3.10	(2.50–3.80)	3.00	(2.50–3.70)	3.35	(2.80–4.30)	0.002
HOMA-IR	1.39	(0.90–2.06)	1.20	(0.84–1.69)	3.20	(2.87–4.04)	<0.0001^||^
TyG	5.88	(4.46–8.12)	5.61	(4.13–7.47)	8.16	(5.88–11.12)	<0.0001^||^
TG/HDL-C	0.77	(0.54–1.24)	0.72	(0.51–1.13)	1.18	(0.70–1.77)	<0.0001^||^

Data reported as count, mean ± SD or median (IQR, expressed as the 25^th^–75^th^ percentile) unless otherwise specified. Insulin resistance categorised as HOMA-IR ≥2.5. Metabolic syndrome categorised according to IDF criteria. Difference between groups (Insulin Sensitive versus Insulin Resistant) assessed using two-tailed independent sample t-tests unless otherwise specified. *Categorical data assessed using chi-squared analysis. ^†^Incomplete data for metabolic syndrome for 35 participants (Insulin sensitive: n = 25; Insulin resistant: n = 10). ^‡^Differences assessed using a two-sample test of proportion. ^§^Incomplete data for physical activity for 9 participants (Insulin sensitive: n = 7; Insulin resistant: n = 2). ^||^Non-parametric data assessed using Mann-Whitney U test. BMI: body mass index. Total-C/HDL-C: Total cholesterol/HDL-cholesterol. HOMA-IR: Homeostatic model assessment for Insulin Resistance. TyG: Triglycerides × glucose. TG/HDL-C: Triglycerides/HDL-cholesterol.

**Figure 1:**
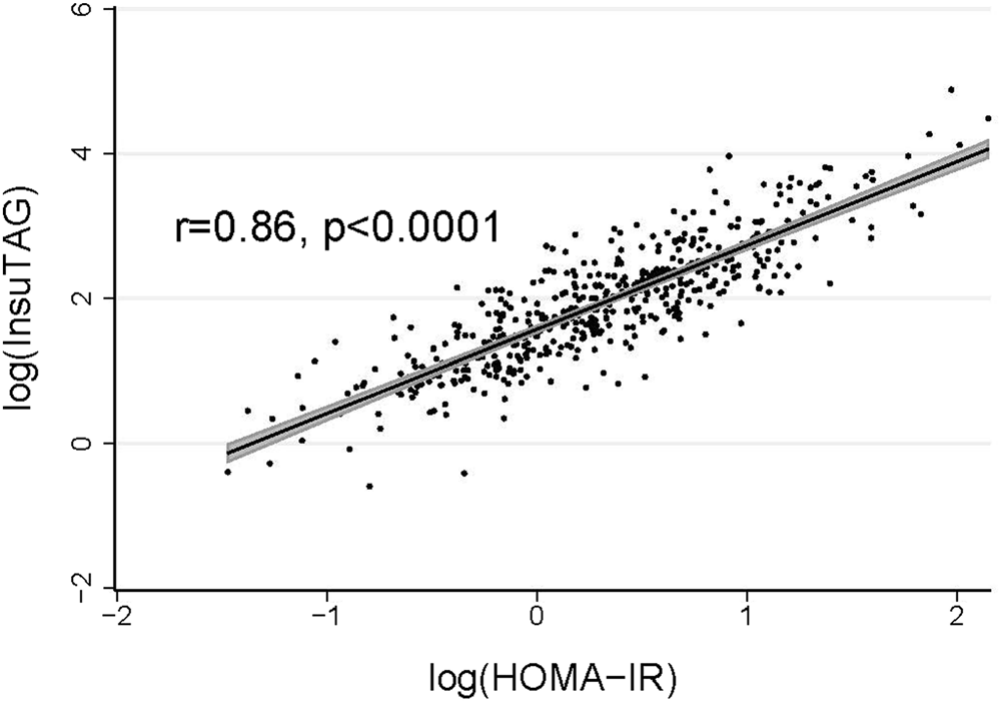
Scatterplot of InsuTAG and HOMA-IR. InsuTAG and HOMA-IR both log_e_ transformed. Solid black line: line of best fit. Grey shaded area: 95% confidence interval.

There is a strong and highly significant positive correlation between InsuTAG and HOMA-IR (r = 0.86; p < 0.0001) (Fig. 1) when compared with the association between HOMA-IR and the other lipid indices, TyG index (triglycerides × glucose) (r = 0.43; p < 0.001) and TG/HDL-C ratio (r = 0.38; p < 0.001) (Table 2). InsuTAG is also positively associated with BMI (r = 0.51, p < 0.001) and WC (r = 0.48, p < 0.001).

**Table 2:** Correlations between contributors to insulin resistance, InsuTAG, and other surrogate markers of insulin resistance.

	Age	BMI	WC	CRP	InsuTAG^†^	HOMA-IR^†^	T:HDLratio	TyG^†^	TG/HDL-C
Age (years)	—	−0.14**	−0.08	−0.01	−0.10*	−0.09*	−0.15***	−0.07	−0.07
BMI (kg/m^2^)	−0.14**	—	0.79***	0.24***	0.51***	0.50***	0.21***	0.35***	0.35***
WC (cm)	−0.08	0.79***	—	0.16***	0.48***	0.47***	0.24***	0.35***	0.40***
CRP (mg/L)	−0.01	0.24***	0.16***	—	0.18***	0.15***	0.12**	0.12**	0.14**
InsuTAG^†^	−0.10*	0.51***	0.48***	0.18***	—	0.86***	0.47***	0.79***	0.75***
HOMA-IR^†^	−0.09*	0.50***	0.47***	0.15***	0.86***	—	0.23***	0.43***	0.38***
Total-C/HDL-C	−0.15***	0.21***	0.24***	0.12**	0.47***	0.23***	—	0.58***	0.73***
TyG^†^	−0.07	0.35***	0.35***	0.12**	0.79***	0.43***	0.58***	—	0.91***
TG/HDL-C^†^	−0.07	0.35***	0.40***	0.14**	0.75***	0.38***	0.73***	0.91***	—

Pearson product-moment correlation coefficients are presented. ^†^Data log_e_ transformed prior to analysis. *p < 0.05, **p < 0.01, ***p < 0.001. BMI: Body Mass Index. WC: Waist Circumference. CRP: C-reactive protein. InsuTAG: Fasting insulin (μIU/L) × triglycerides (mmol/L). HOMA-IR: Fasting glucose (mmol/L) × insulin (μIU/L). Total-C/HDL-C: Total cholesterol (mmol/L)/HDL cholesterol (mmol/L). TyG: Fasting triglycerides (mmol/L) × glucose (mmol/L). TG/HDL-C: Fasting triglycerides (mmol/L)/HDL-cholesterol (mmol/L).

Multiple regression analyses were used to evaluate the relationships between InsuTAG and other surrogate markers of IR with HOMA-IR (Table 3). Model 1 presents the unadjusted analyses. Model 2 present the analyses adjusted for age, gender, WC and C-reactive protein (CRP). All surrogate markers of IR were significant predictors of HOMA-IR, both independently and after adjusting for covariates (p < 0.0001), however, InsuTAG accounted for a greater proportion of variability in HOMA-IR (Model 1, R^2^ = 0.739; Model 2, R^2^ = 0.738) than did the TyG index (Model 1, R^2^ = 0.183; Model 2, R^2^ = 0.303) or the TG/HDL-C ratio (Model 1, R^2^ = 0.145; Model 2, R^2^ = 0.284). Evaluation of the partial correlations indicated that, after adjusting for covariates, InsuTAG accounted for 65.0% of the variability in HOMA-IR scores, whereas TyG and TG/HDL-C were independently associated with only 7.9% and 5.4% of the change in HOMA-IR scores, respectively. Examination of the regression coefficient revealed that, after adjusting for covariates, a 10% increase in InsuTAG resulted in a corresponding increase of 2.57% in HOMA-IR (p < 0.0001). In addition, logistic regression showed that after adjusting for age, gender, WC and CRP, each one-unit increase in InsuTAG increased the odds of having IR by 20% (OR (95%CI): 1.20 (1.15–1.26), p < 0.0001) and MetS by 16% (OR (95%CI): 1.16 (1.11–1.21), p < 0.0001).

**Table 3:** Regression Models for predicting Insulin Resistance (IR)*.

Surrogate Markers of IR	Model 1					Model 2				
	*Model Coefficients*		*Model Statistics*		β-coefficient	*Model Coefficients*		p	*Model Statistics*	
	β-coefficient	p	adj. *R* ^2^	p		Partial R^2^	Semi-Partial R^2^		adj. *R* ^2^	p
**InsuTAG**			0.739	<0.0001					0.738	<0.0001
InsuTAG*	0.637	<0.0001			0.613	0.650	0.487	<0.0001		
Age	—	—			0.0002	<0.0001	<0.0001	0.930		
Gender	—	—			−0.022	0.0009	0.0002	0.524		
WC	—	—			0.003	0.0077	0.002	0.058		
CRP	—	—			0.00005	<0.0001	<0.0001	0.988		
**TyG**			0.183	<0.0001					0.303	<0.0001
TyG*	0.559	<0.0001			0.342	0.079	0.060	<0.0001		
Age	—	—			−0.003	0.002	0.001	0.406		
Gender	—	—			0.176	0.022	0.016	0.001		
WC	—	—			0.020	0.149	0.120	<0.0001		
CRP	—	—			0.007	0.003	0.002	0.214		
**TG/HDL-C Ratio**			0.145	<0.0001					0.284	<0.0001
TG/HDL-C*	0.380	<0.0001			0.217	0.054	0.041	<0.0001		
Age	—	—			−0.003	0.001	0.001	0.426		
Gender	—	—			0.214	0.032	0.024	<0.0001		
WC	—	—			0.021	0.157	0.132	<0.0001		
CRP	—	—			0.006	0.003	0.002	0.260		

*IR defined by HOMA-IR value, InsuTAG, TyG and TG/HDL-C log_e_ transformed prior to analysis. Model 1: Unadjusted estimates. Model 2: Adjusted for age, gender, waist circumference, CRP. A 1% increase in InsuTAG corresponds with a 0.265% increase in HOMA-IR or a 10% increase in InsuTAG corresponds with an increase to HOMA-IR of 2.57%.

**Figure 2:**
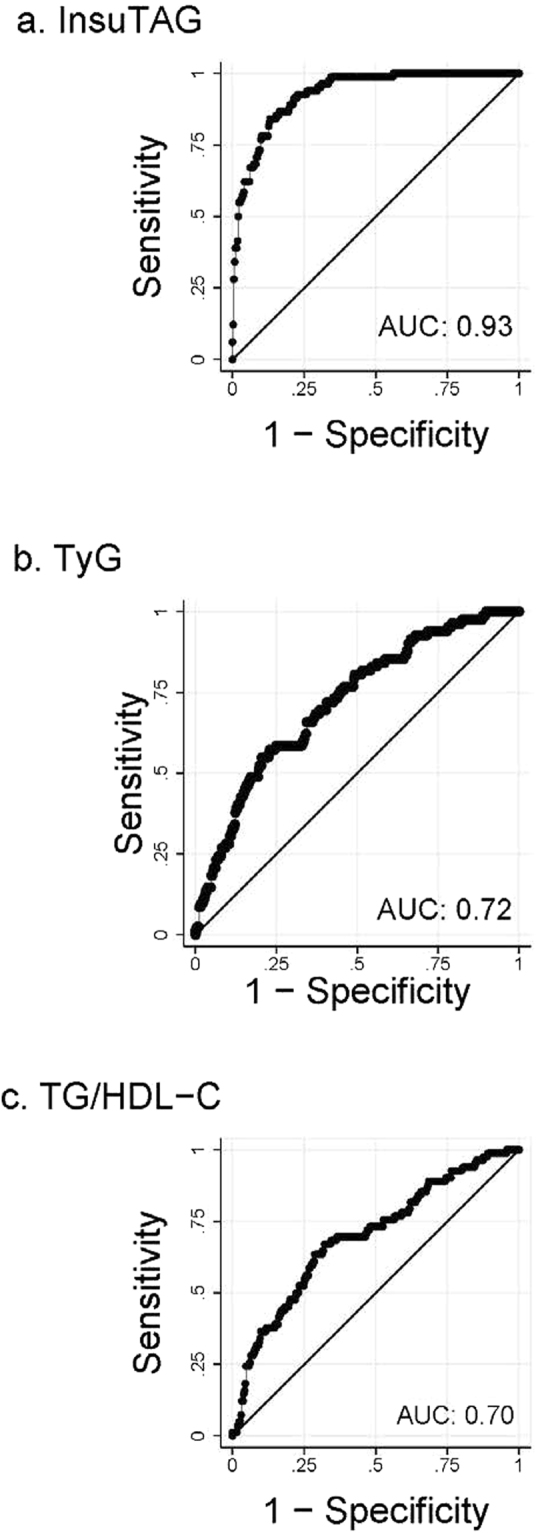
Receiver operating characteristic (ROC) curves for identifying Insulin Resistance (IR) using surrogate markers of IR. IR categorised as HOMA-IR ≥2.5. ROC curve for InsuTAG (**a**); ROC curve for TyG (**b**); ROC curve for TG/HDL-C (**c**). AUC: Area under the curve.

**Figure 3:**
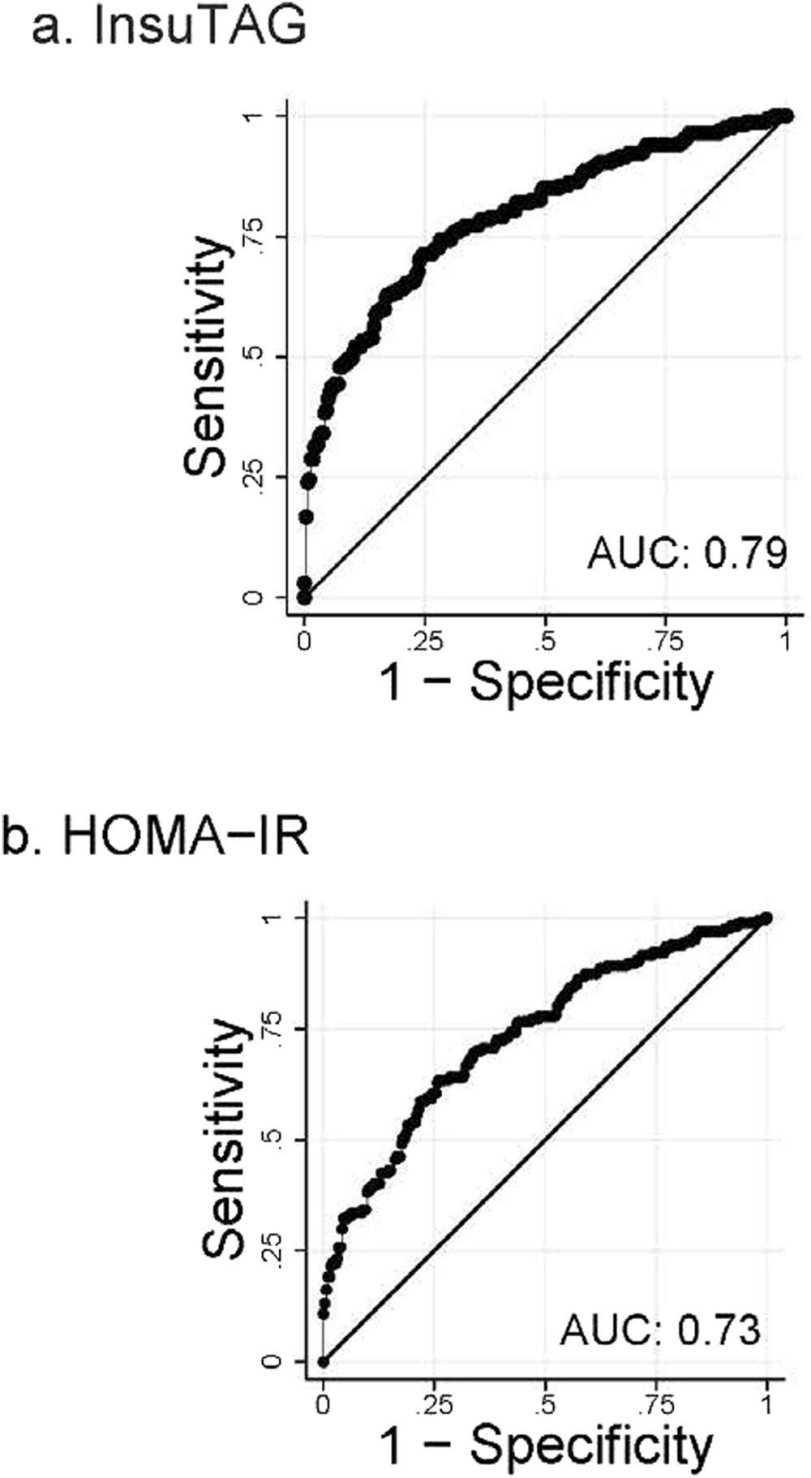
Receiver operating characteristic (ROC) curves for identifying Metabolic Syndrome (MetS) using InsuTAG and HOMA-IR. MetS categorised according to IDF criteria. ROC curve for InsuTAG(**a**); ROC curve for HOMA-IR (**b**). AUC: Area under the curve.

ROC curves of InsuTAG and surrogate markers of IR were plotted to compare the predictive values for IR and MetS (Fig. 2). InsuTAG had significantly greater Area under the curve (AUC) values (AUC = 0.93, p < 0.001) than TyG index (AUC = 0.72) or TG/HDL-C (AUC = 0.70) for the identification of IR. For the identification of MetS, the AUC of InsuTAG (0.79) was significantly higher than HOMA-IR (0.73, p = 0.001) (Fig. 3), fasting insulin (0.69, p = 0.000) and WC (0.72, p = 0.009). The optimal cut off value for InsuTAG in identifying IR in the current study population was determined to be 11.2, with a sensitivity of 84.15% and specificity of 86.88%. At this cut-off, the sensitivity and specificity for MetS was 49.70% and 90.49% respectively (Table 4). Positive and negative predictive values along with likelihood ratios are also presented in Table 4. After determining the cut-off values for InsuTAG, the study population were categorised into two groups: InsuTAG < 11.2 (n = 364), and InsuTAG ≥11.2 (n = 122). 74.34% of participants with InsuTAG values ≥11.2 were identified with MetS compared to only 24.56% with InsuTAG values < 11.2 (Table 5, p < 0.0001). All the key components of the metabolic syndrome, WC (104.65 ± 12.45 vs 94.51 ± 11.79 cm); fasting glucose (5.46 ± 0.51 vs 5.14 ± 0.54 mmol/L); fasting TG [1.87 (1.43–2.31) vs 1.01 (0.79–1.30) mmol/L]; and Total-C/HDL-C [3.60 (3.00–4.70) vs 2.90 (2.40–3.50)] were significantly (p < 0.0001) higher in participants with InsuTAG values above the cut-off compared with participants InsuTAG values below the cut-off (Table 5). BMI, diastolic blood pressure, fasting insulin and HOMA-IR values were also significantly higher in the InsuTAG ≥11.2 group, whereas systolic blood pressure, glycosylated haemoglobin (HbA1c), total cholesterol and low density lipoprotein-cholesterol (LDL-C) did not differ between the two groups (Table 5). The lipid index values, TyG [10.29 (7.69–12.60) vs 5.25 (4.00–6.54)] and TG/HDL-C [1.41 (1.07–1.91) vs 0.66 (0.48–0.89)] were almost doubled in participants with InsuTAG values above the cut-off.

**Table 4:** Predictive values of proposed InsuTAG cut-off of 11.2 for the identification of Insulin Resistance and Metabolic Syndrome.

	Sensitivity	Specificity	PV+	PV−	LR+	LR−
Insulin Resistance	84.15%	86.88%	0.56	0.96	6.41	0.18
Metabolic Syndrome	49.70%	90.49%	0.75	0.75	5.23	0.56

Insulin resistance classified according to HOMA-IR ≥2.5. Metabolic Syndrome classified according to IDF criteria. PV+: Positive predictive value. PV−: Negative predictive value. LR+: Positive likelihood ratio. LR−: Negative likelihood ratio.

**Table 5:** Participant characteristics and metabolic parameters of participants stratified into subgroups according to the proposed InsuTAG cut-off of 11.2.

	InsuTAG < 11.2 n = 364		InsuTAG ≥11.2 n = 122		*p*-value
**Gender (n, (%))**					
Male	153	(42.03)	50	(40.98)	0.839*
Female	211	(57.97)	72	(59.02)	0.839*
Age (years)	78.18	±7.20	76.57	±6.94	0.031
Waist Circumference (cm)	94.51	±11.79	104.65	±12.45	<0.0001
Height (m)	1.63	±0.09	1.62	±0.10	0.762
Weight (kg)	71.62	±13.43	82.57	±14.87	<0.0001
BMI (kg/m^2^)	26.96	±4.21	31.25	±4.36	<0.0001
Metabolic Syndrome^†^ (n, (%))	84	(24.56)	83	(74.34)	<0.0001*
Dietary Energy Intake (Cal/day)	1959	±732	2008	±705	0.516
Protein (g/day)	90.98	±36.30	90.36	±31.80	0.868
Fat (g/day)	68.89	±32.59	69.10	±29.75	0.951
Saturated Fat (%Energy/day)	10.73	±3.41	10.59	±2.68	0.671
Fibre (g/day)	31.68	±14.80	32.18	±15.22	0.749
**Physical Activity** ^**‡**^ **(n, (%))**					
High	95	(26.61)	25	(20.83)	0.203^§^
Moderate	194	(54.34)	69	(57.50)	0.544^§^
Low	55	(15.41)	19	(15.83)	0.912^§^
Sedentary	13	(3.64)	7	(5.83)	0.269^§^
Systolic Blood Pressure (mmHg)	145.22	±21.25	148.49	±19.63	0.183
Diastolic Blood Pressure (mmHg)	74.03	±9.77	76.64	±9.26	0.021
Fasting Glucose (mmol/L)	5.14	±0.54	5.46	±0.51	<0.0001
Fasting Insulin (μIU/L)	5.0	(3.7–6.9)	11.3	(8.5–14.4)	<0.0001^||^
HbA1c (%)	5.71	±0.32	5.76	±0.39	0.213
HbA1c (mmol/mol)	39	±3.20	39	±4.30	0.213
Total Cholesterol (mmol/L)	4.66	±1.00	4.78	±1.07	0.269
Triglycerides (mmol/L)	1.01	(0.79–1.30)	1.87	(1.43–2.31)	<0.0001^||^
LDL-C (mmol/L)	2.56	±0.89	2.63	±0.97	0.443
HDL-C (mmol/L)	1.57	(1.32–1.82)	1.26	(1.08–1.52)	<0.0001^||^
Total-C/HDL-C	2.90	(2.40–3.50)	3.60	(3.00–4.70)	<0.0001
HOMA-IR	1.15	(0.82–1.62)	2.67	(2.05–3.55)	<0.0001^||^
TyG	5.25	(4.00–6.54)	10.29	(7.69–12.60)	<0.0001^||^
TG/HDL-C	0.66	(0.48–0.89)	1.41	(1.07–1.91)	<0.0001^||^

Data presented as mean ± SD or median (IQR, expressed as the 25^th^–75^th^ percentile) unless otherwise specified. Metabolic Syndrome categorised according to IDF criteria. Differences between groups (InsuTAG <11.2 versus InsuTAG ≥11.2) assessed using two-tailed independent sample t-test unless otherwise specified. *Differences assessed using chi-squared analysis. ^†^Incomplete data for metabolic syndrome for 35 participants (InsuTAG <11.2: n = 26; InsuTAG ≥11.2: n = 9). ^‡^Incomplete data for physical activity for 9 participants (InsuTAG <11.2: n = 7; InsuTAG ≥11.2 n = 2). ^§^Differences assessed using a two-sample test of proportion. ^||^Non-parametric data assessed using Mann-Whitney U test. BMI: body mass index. Total-C/HDL-C: Total cholesterol/HDL-cholesterol. HOMA-IR: Homeostatic model assessment for Insulin Resistance. TyG: Triglycerides × glucose. TG/HDL-C: Triglycerides/HDL-cholesterol.

## Discussion

Many studies have evaluated lipid ratios, homeostatic models and individual metabolic variables for predicting IR^6,22,23^, however, none account for fasting insulin and fasting TG in a single model or formula. The current study has evaluated the use of InsuTAG, a novel marker derived from the product of two key continuous variables, fasting insulin and fasting TG, as a predictor for IR and metabolic abnormalities. InsuTAG demonstrated a stronger positive association (0.86) with HOMA-IR than the other individual metabolic markers and lipid surrogate markers analysed in this study. InsuTAG, TyG and TG/HDL-C were all independent predictors of IR in regression models; InsuTAG was the highest (65.0%) contributor to prediction of IR in the study population. ROC analysis also indicated InsuTAG (AUC 0.93) was the favourable marker over TyG index and TG/HDL-C for predicting IR.

HOMA-IR is the most common or frequently used index for assessing IR and closely mirrors HEC values^24^. Given that it is not feasible to conduct HEC in large study populations, in this study HOMA-IR scores of ≥2.5 were used to identify IR. Previous studies proposed surrogate lipid-based markers such as TyG index and TG/HDL-C ratio as an alternate approach to predict IR^6,25^. Consistent with previous reports^22,26,27^, the current study found significant and positive associations between HOMA-IR and BMI, WC and lipid ratios. InsuTAG demonstrated a strong positive association with HOMA-IR, representing a close association with IR, higher than that of other lipid-based surrogate markers and anthropometric measurements analysed in this study population. Multivariate regression analysis indicated that both TyG index and TG/HDL-C were independent predictors for IR, findings that are consistent with previous published studies^7,22^. Amongst these independent predictors, InsuTAG accounted for the greatest variance in IR for this study population, accounting for 73.9% (without adjusting for covariates) and 65% (after adjusting for covariates) of variance in HOMA-IR. Along with the benefit of accounting for two key factors involved in the development of IR, these results indicate that InsuTAG may be a reliable and physiologically relevant marker for IR that can be easily calculated from routine clinical investigations. However, as HOMA-IR is not a gold standard method of determining IR, further validation studies of the predictive capacity of InsuTAG using clamp study insulin sensitivity index values are required.

AUC values from the ROC analysis showed that InsuTAG represented 93% probability of identifying individuals with IR in this study population, comparatively greater than the AUC’s of other lipid markers. The high sensitivity and specificity values of InsuTAG and positive likelihood ratio of 6.41 (Table 4) provides reasonable justification to explore InsuTAG as a diagnostic tool for IR. ROC analysis identified 11.2 as the optimal cut-off value for predicting IR in this study population. The corresponding value for identification of MetS was 8.0. The AUC for InsuTAG (0.79) suggests it is higher to HOMA-IR (0.73), fasting insulin (0.69) and WC (0.72) for predicting MetS in this study population. Metabolic characteristics of participants with InsuTAG scores above and below the suggested cut-off were compared. Significant differences in WC, BMI, fasting glucose, fasting insulin, TG, HDL-C and blood pressure, all of which are key components of MetS, were observed. There was no significant difference in participant characteristics between sub-groups stratified by the InsuTAG cut-off values for predicting IR (11.2) and MetS (8.0), other than minor changes in sensitivity and specificity values for MetS. No further conclusions on these associations can be made at this point warranting further exploration in prospective studies to determine whether InsuTAG alone can predict the development of MetS.

We do acknowledge some limitations to this study. The study population is older and predominantly Caucasian, limiting the generalisability of InsuTAG to younger people and other ethnicities. Since it is less feasible to conduct HEC in studies with larger sample sizes, InsuTAG values were compared with HOMA-IR values rather than insulin sensitivity index values from clamp studies. Additional exploration of InsuTAG using glucose clamp studies is required to further validate this marker. Furthermore, because of the cross-sectional design of this study, no causal relationships can be determined. Longitudinal studies are required to evaluate whether InsuTAG can predict development of IR and MetS.

Surrogate markers for IR are less invasive and closely mirror correlation with HEC for metabolic and CVD risk. A recently published paper ^(20)^ on correlation of surrogate indices with HEC, concluded that surrogate markers including fasting insulin provided the most information relating to IR, compared with other complex and invasive procedures. The combination of fasting insulin and an indicator of reduced lipid clearance could provide more reliable information on IR and metabolic abnormalities.

In conclusion, we have proposed and evaluated a novel marker for IR that accounts for both fasting insulin and TG. It is simple to calculate and feasible for large cohort studies. This study substantiates and shows InsuTAG as a predictor of IR and a predictor of metabolic syndrome with higher sensitivity and specify values over other anthropometry and existing lipid surrogate indices. Further research is required to validate InsuTAG against HEC and determine whether it can accurately predict the development of IR and MetS in prospective studies.

## Methods

This study is a sub-study of the Retirement Health and Lifestyle (RHLS), a cross-sectional study of Australians aged 65 years and older, living in the Central Coast of New South Wales, Australia. Inclusion and exclusion criteria for participants in RHLS study has been described in detail elsewhere^28,29^. In brief, participants were invited to participate in the RHLS if they were: aged between 65 years or over; living in the Wyong Shire or Gosford City local government areas and living independently in retirement villages or within the community. Participants were included in the present study if: their diabetic status could be determined; their plasma fasting glucose, insulin and TG levels were available; and if they were not taking any TG-lowering medication. Participants provided written informed consent and ethics approval for this study was obtained from the University of Newcastle Human Research Ethics Committee (reference no. H-2008-0431) and the Northern Sydney Central Coast Health Human Research Ethics Committee (reference no. 1001–031 M). All testing was performed in accordance with the approved guidelines.

Demographic information, medical history, and information relating to medication and supplement intake were obtained from participants via interviewer-administered and self-administered questionnaire. Height and weight of study participants were measured using a portable stadiometer (design no. 1013522 Surgical and Medical products) and digital scales (Tanita HD 316 or Wedderburn UWPM150). BMI was calculated using the standard formula [weight (kg)/height (m)^2^]. WC was measured with a non-elastic measuring tape at the midpoint between the iliac crest and coastal margin in the mid-auxiliary line. All anthropometric measurements were conducted by trained research officers.

Information on dietary intake was obtained from study participants using a self-administered semi-quantitative food frequency questionnaire (FFQ) adapted from a validated Commonwealth Scientific and Industrial Research Organisation Human Nutrition FFQ^30^. Diet, energy and nutrient intake information was analysed using Food Works Professional (2009 edition, version 6.0.2562, Xyris software, Brisbane, Australia) in association with the following databases; Australia (fatty acids), Abbott products, AusFoods (brand) 2006, AusNut (all foods) 2007, and the New Zealand Vitamin and Mineral Supplement 1999.

Physical activity was assessed during the interviewer-administered questionnaire using questions designed to capture the frequency, duration and intensity of physical activity undertaken during the previous seven days. The questions were adapted from validated questionnaires measuring physical activity, and captured both incidental (e.g. household chores, gardening) and intentional (e.g. recreational sports, strength training) physical activity. Blood pressure measurements were taken with an OMRON 1A2 digital automatic blood pressure monitor in accordance with the “Measuring Blood Pressure” protocol published by the National Heart Foundation in 2008.

Blood samples were collected by trained phlebotomists following an overnight fast of at least 10 hours. Blood glucose control related parameters [fasting glucose (mmol/L), fasting insulin (mIU/L) and HbA1c], lipid profile [TG, total cholesterol, LDL-C and HDL-C; mmol/L] and CRP (mg/mol) were analysed by Hunter Area Pathology Service using standard laboratory procedures. Fasting insulin and glucose were used to measure IR using HOMA-IR scores (fasting insulin × fasting glucose/22.5). Study participants with HOMA-IR values ≥2.5 were categorised as insulin resistant. TyG index (fasting glucose × fasting TG) and TG/HDL-C ratio were also assessed. InsuTAG was calculated by multiplying fasting insulin (mU/L) and fasting TG (mmol/L). Participants were categorised as having MetS according to IDF criteria for abdominal obesity (waist circumference ≥94 cm for males, or ≥80 cm for females) plus any two of the following conditions (or self-report of receiving treatment for any of those conditions): high TG (≥1.7 mmol/L); high fasting glucose (≥5.6 mmol/L); high BP (≥130 mmHg systolic or ≥85 mmHg diastolic); and/or low HDL-C (<1.03 mmol/L for males, <1. 29 mmol/L for females).

The data of all variables included in the analysis were tested for normality using histograms and Shapiro-Wilk’s tests and are presented as mean ± SD or median (IQR) as appropriate. Heavily skewed parameters were log-transformed (log base e) prior to correlation and regression analyses. Distributions were reassessed after transformation and in all instances the log transformation successfully achieved a normal distribution. Bivariate relationships between continuous variables were assessed using Pearson’s Product-moment correlation. Standard multiple regression was used to assess the relationship of HOMA-IR with InsuTAG and other surrogate markers of IR, with and without adjustment for the potentially confounding variables: age, gender, WC and blood levels of CRP. Logistic regression was used to determine whether an increase in InsuTAG score was associated with increased odds of having IR or MetS. Receiver operating characteristic (ROC) curves were constructed for InsuTAG and other surrogate markers of IR to assess whether they were effective in identifying either IR and/or MetS. For each marker, the area under the curve (AUC) was compared against HOMA-IR using the Stata command *roccomp*. Youden’s index was used to determine the optimum cut-off point, the point which has the greatest sensitivity and specificity, Participants were categorised according to whether their InsuTAG scores fell below or above the suggested cut-off point. Group differences were assessed using independent sample t-tests, Mann-Whitney U tests, or chi-squared analysis as appropriate. Significance was set at p < 0.05. All statistical analyses were conducted using Stata version 14.1 (StataCorp, Texas, USA).
